# Association between polymorphisms in the promoter region of the apolipoprotein E (APOE) gene and Alzheimer's disease: A meta-analysis

**DOI:** 10.17179/excli2017-289

**Published:** 2017-06-20

**Authors:** Hanyan Xiao, Yifeng Gao, Lei Liu, Yan Li

**Affiliations:** 1 Department of Neurology, Second Affiliated Hospital of Mudanjiang Medical University, Mudanjiang 157010, Heilongjiang, China; 2 Department of Respiratory Medicine, Second Affiliated Hospital of Mudanjiang Medical University, Mudanjiang 157010, Heilongjiang, China; 3 Department of Gastroenterology, Second Affiliated Hospital of Mudanjiang Medical University, Mudanjiang 157010, Heilongjiang, China; 4 Mudanjiang Medical University Affiliated HongQi Hospital, Mudanjiang 157011, Heilongjiang, China, Department of General Surgery

**Keywords:** Alzheimer's disease, apolipoprotein E, promoter region, polymorphism

## Abstract

Several studies have evaluated the role of polymorphisms in the promoter region of *APOE* gene that encodes apolipoprotein E (*APOE*) and the susceptibility to Alzheimer's disease (AD). The aim of this literature review and meta-analysis was to investigate the relationship between the *APOE* promoter region single nucleotide polymorphisms (SNPs) (rs449647, -491A/T; rs769446, -427T/C and rs405509 -219T/G) and the risk of developing AD. Eligible controlled studies published up to November 2016 were retrieved from main online scientific and medical databases. Odds ratio (OR) and 95 % confidence interval (CI) were used to calculate the strength of the relationship. A total of 23 publications (19 for rs449647, ten for rs769446 and ten for rs405509) were retrieved that included 5,703 patients with AD and 5,692 controls. The C allele of the rs769446 variant of the promoter region of *APOE* gene was significantly associated with an increase of risk of AD (OR = 1.271, 95 % CI = 1.114-1.449, *P* < 0.0001), while other genetic models of this variant were not related with susceptibility to AD. Rs449647 and rs405509 polymorphisms of *APOE* gene were not associated with an increase of risk of AD. The findings of this literature review and meta-analysis have shown that rs769446 polymorphism in the promoter region of *APOE* gene could be a risk factor for AD. Future large-scale studies on the role of polymorphisms in the promoter region of *APOE* gene in AD are still awaited.

## Introduction

Alzheimer's disease (AD) is a chronic and progressive neurodegenerative disease that usually starts slowly and becomes more severe over time (Ballard et al., 2011[7]; Vinters, 2015[53]). Worldwide, AD is now the leading cause of dementia in late adult life, accounting for 60 % to 70 % of all cases of dementia (Mayeux and Stern, 2012[35]). The most common symptoms include cognitive impairment, loss of short-term memory, problems with language, disorientation, mood swings, loss of motivation, and behavioral issues (Geda et al., 2013[18]). According to the 2016 data from the Alzheimer's Association, an estimated 5.4 million Americans have AD, and approximately 700,000 Americans age ≥ 65 years will die with this disease in 2016 (Alzheimer's Association, 2016[4]). Furthermore, by 2050, one new case of Alzheimer's is expected to develop every 33 seconds, resulting in nearly 1 million new cases per year (Reitz and Mayeux, 2014[41]; Sosa-Ortiz et al., 2012[46]). 

However, the etiology of AD remains unclear. Although there are now several drugs that can be used in the treatment of AD, randomized controlled clinical trials have yet to show robust treatment benefits (Gandy and Dekosky, 2013[16]). Also, the study of the long-term effect of any intervention for patients with AD is a great methodological challenge (Wimo, 2015[56]). Therefore, there is an urgent need to identify biomarkers that can predict this disease and guide therapeutic strategies. 

AD is a heterogeneous disease, and about 70 % of the risk is believed to be genetic with many genes involved (Karch et al., 2014[24]). The *APOE* gene that encodes apolipoprotein E (ApoE) located on human chromosome 19 is a multifunctional protein and is a major cholesterol carrier with central roles in lipid metabolism, neurobiology, and neurodegenerative diseases (Carrasquillo et al., 2013[11]; Hatters et al., 2006[20]). The *APOE* gene has been recognized as a possible genetic risk factor for the onset of AD (Yu et al., 2014[60]). There are known to be three isoforms (ε2, ε3, and ε4) with different effects on lipid and neuronal homeostasis (Phillips 2014[39]). Epidemiologic studies have identified that these isoforms are the main genetic determinants of the progression of AD (Holtzman et al., 2012[21]). For example, individuals carrying the ε4 allele are at increased risk of AD when compared with those carrying the more common ε3 allele, whereas the ε2 allele decreases risk (Liu et al., 2013[33]). In addition to the polymorphism within the coding region, uncovering the polymorphisms within the *APOE* promoter region may also be beneficial to predict AD risk. 

The *APOE* gene encodes apolipoprotein E (*APOE*) and has a promoter region that is complex, with several regulatory elements located in the proximal 5' flanking region and the first intron of the human *APOE* gene (Berg et al., 1995[9]; Garcia et al., 1996[17]). Thus, polymorphisms in this region could have functional repercussions mediated by the regulation of *APOE* gene transcription. Three single nucleotide polymorphisms (SNPs), at -491 (A/T, rs449647), -427 (T/C, rs769446) and -219 (T/G, rs405509) upstream from the +1 transcription start site have been identified. These SNPs might influence *APOE* transcriptional activity through differential binding of transcription factors (Sala Frigerio and De Strooper 2016[43]). Although several studies have estimated the effect of *APOE* gene variants in the promoter region on AD susceptibility, the results remain inconsistent. Therefore, we conducted a systematic review of the literature and meta-analysis to evaluate the current status of the association between polymorphisms in the promoter region of the *APOE *gene and the risk of AD.

## Materials and Methods

### Identification of eligible studies

A comprehensive literature search was conducted using the online databases of PubMed, Web of Science, Embase, Wanfang, and CNKI (China National Knowledge Infrastructure) to retrieve relevant articles published until November 2016. The Medical Subject Heading (MeSH) terms were: “Alzheimer's disease” or “Alzheimer” and “apolipoprotein E” or “APOE,” and “polymorphism” or “single nucleotide polymorphism” or “single nucleotide variant”. 

The corresponding Chinese characters were used in the Chinese databases. References were manually checked to obtain more relevant articles. Studies written in English or in Chinese language were included. When the same authors or laboratories published more than one article that included the same study participants, only the most complete study was included into the meta-analysis.

### Criteria for article screening

The eligible articles had to meet the following inclusion criteria: 1) case-control studies evaluating the relationship between polymorphisms in the promoter region of the *APOE* gene and susceptibility to Alzheimer's disease (AD); 2) diagnosis of patients with AD based on standards of the National Institute of Neurological and Communicative Disorders and Stroke (NINCDS) or Alzheimer's Disease and Related Disorders Association (ADRDA) working group (McKhann et al., 1984[36]); 3) inclusion of age-matched controls of unrelated participants without dementia; 4) results that were expressed as an odds ratio (OR) with corresponding 95 % confidence interval (95 % CI); and 5) distribution of the genotype of controls for each polymorphism were required to be in Hardy-Weinberg equilibrium (HWE). 

The exclusion criteria were: 1) review article or conference papers; 2) studies without a control group; 3) studies with duplicated data; 4) inability to extract the study data; and 5) a study population that included patients with dementia due to causes other than AD.

### Quality assessment and data extraction

Two authors independently assessed the quality of the extracted publications based on the study inclusion and exclusion criteria. Any disagreement was resolved by discussion with the third author, with each item discussed until a final consensus was reached. The following information was retrieved: name of the first author, publication year, country, mean patient age, number of cases and controls, distribution of genotypes, diagnostic criteria of AD, sources of controls, and genotyping methods.

### Statistical analysis

Statistical analysis was carried out using the Stata 12.0 statistical software package (StataCorp, USA). The relationship between the *APOE* gene promoter polymorphisms and the risk of AD were evaluated by odds ratio (OR) with 95 % confidence interval (95 % CI). The significance of the pooled OR was determined by the Z-test. A *P*-value less than 0.05 was considered to be significant. 

For each genetic variant, the allelic model, the homogeneous model, the heterogeneous model, the domain model, and the recessive model were calculated. Cochran's Q test and the I^2^ test were employed to evaluate the heterogeneity of the included articles. The fixed-effect model was used when the P-value of Cochran's Q test was more than 0.10, and I^2^ of the I^2^ test was less than 50 %; otherwise, the random-effect model was used. To assess whether our results were substantially influenced by the presence of any individual study, we conducted a sensitivity analysis by systematically omitting each study and recalculating the significance of the result. Egger's test and Begg's test were performed to examine the publication bias. 

## Results

### Characteristics of eligible studies

We first identified 500 relevant publications on *APOE* gene polymorphisms and the risk of Alzheimer's disease (AD). However, after applying the study inclusion and exclusion criteria, a total of 23 publications were finally included for study and included 5,703 patients with AD, and 5,692 controls. 

**Figure 1:**
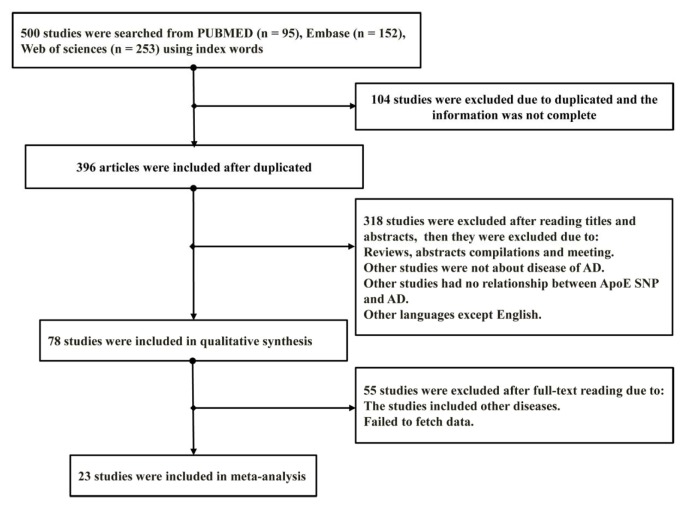
Flow chart of publication selection process in literature review and meta-analysis

Figure 1(Fig. 1) shows the selection process of this meta-analysis. The 23 articles included studies that were conducted in 16 countries: three in USA (Nicodemus et al., 2004[37]; Parker et al., 2005[38]; Tycko et al., 2004[50]); two in China (Wang et al., 2017[55]; Yang et al., 2003[59]), two in Tunisia (Achouri-Rassas et al., 2014[2], 2015[1]), one in France (Verpillat, 2000[52]), two in Italy (Bizzarro et al., 2009[10]; Licastro et al., 1999[31]), two in Japan (Kimura et al., 2000[26]; Toji et al., 1999[48]), two in the UK (Belbin et al., 2007[8]; Lambert et al., 1998[27]), one in Canada (Song et al., 1998[45]), one in Australia (Laws et al., 1999[30]), one in Spain (Alvarez-Arcaya et al., 2001[3]), one in the Netherlands (Roks et al., 2002[42]), one in Hungary (Juhász et al., 2005[23]), one in Brazil (Bahia et al., 2008[6]), one in Slovakia (Trebuňová et al., 2009[49]), one in Korea (Chung et al., 2013[13]), and one in Poland (Limon-Sztencel, 2016[32]).

Three polymorphic sites in the promoter region of *APOE* gene were reported: rs449647 (-491A/T), rs769446 (-427T/C) and rs405509 (-219T/G). All specimens were from blood samples. The sample size ranged from 105 to 1082. The genotype distributions in the controls of all studies were in agreement with the Hardy-Weinberg equilibrium (HWE). The main characteristics of included studies are presented in Table 1(Tab. 1) (References in Table 1: Song et al., 1998[45]; Lambert et al., 1998[27]; Licastro et al., 1999[31]; Toji et al., 1999[48]; Laws et al., 1999[30]; Zurutuza et al.; 2000[61]; Kimura et al., 2000[26]; Alvarez-Arcaya et al., 2001[3]; Roks et al., 2002[42]; Yang et al., 2003[59]; Nicodemus et al., 2004[37]; Tycko et al., 2004[50]; Parker et al., 2005[38]; Juhász et al., 2005[23]; Belbin et al., 2007[8]; Bahia et al., 2008[6]; Trebuňová et al., 2009[49]; Chung et al., 2013[13]; Bizzarro et al., 2009[10]; Achouri-Rassas et al., 2014[2]; Wang et al., 2017[55]; Achouri-Rassas et al., 2015[1]; Limon-Sztencel, 2016[32]).

### Association between rs449647 (-491A/T) APOE gene polymorphism and the risk of AD 

There were 19 published studies that included the rs449647 (-491A/T) *APOE* gene polymorphism and the risk of AD. Between-study heterogeneity was found, and the random-effect model was employed to synthesize these data. Summary odds ratios (ORs) and tests for heterogeneity of published studies are shown in Table 2(Tab. 2). 

The T allele of rs449647 polymorphism was not associated with increased risk of AD when compared with the A allele (T vs. A: OR = 0.962, 95 % CI = 0.742-1.247, *P* = 0.769). This non-significant relationship was observed in other genetic models as well (TT vs. AA: OR = 1.11, 95 % CI = 0.891-1.382, *P* = 0.352; TT vs. AT: OR = 1.02, 95 % CI = 0.699-1.49, *P* = 0.918; AT+TT vs. AA: OR = 0.941, 95 % CI = 0.723-1.225, *P* = 0.651; TT vs. AA+AT: OR = 0.928, 95 % CI = 0.583-1.478, *P* = 0.753). 

**Figure 2:**
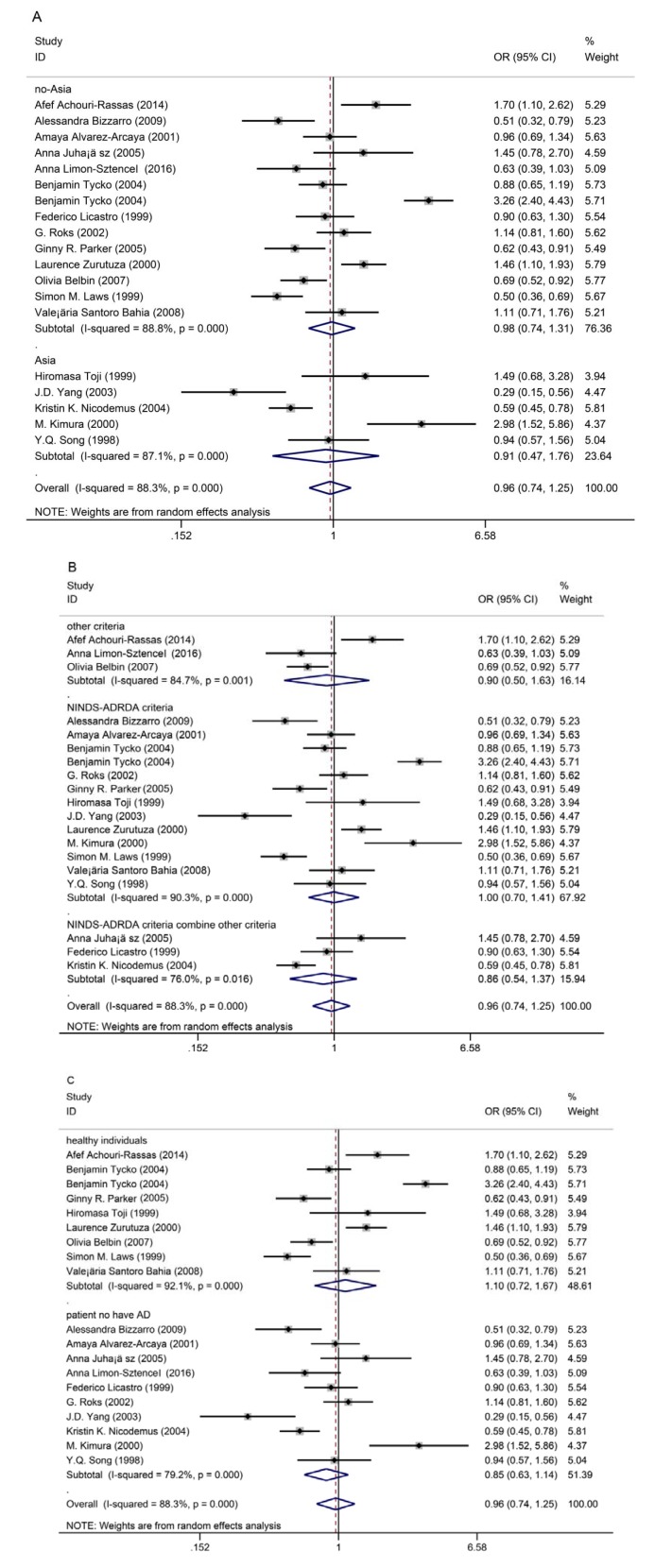
Subgroup analysis of the association between rs449647 polymorphism and susceptibility to Alzheimer's disease (AD) A: ethnicity; B: diagnostic criteria of patients with AD; C: participants in the control groups

Subgroup analysis by ethnicity showed that there was a significant difference between Asian and non-Asian patients with AD and effects of rs449647 polymorphism and AD risk (*P* < 0.0001), as shown in Figure 2A(Fig. 2) (References in Figure 2: Achouri-Rassas et al., 2014[2]; Bizzarro et al., 2009[10]; Alvarez-Arcaya et al., 2001[3]; Juhász et al., 2005[23]; Limon-Sztencel, 2016[32]; Tycko et al., 2004[50]; Licastro et al., 1999[31]; Roks et al., 2002[42]; Parker et al., 2005[38]; Zurutuza et al., 2000[61]; Belbin et al., 2007[8]; Laws et al., 1999[30]; Bahia et al., 2008[6]; Toji et al., 1999[48]; Yang et al., 2003[59]; Nicodemus et al., 2004[37]; Kimura et al., 2000[26]; Song et al., 1998[45]).

Subgroup analysis by diagnostic criteria for patients with AD showed that there was a significant difference for selection criteria in each study, as shown in Figure 2B(Fig. 2).

Subgroup analysis by participants in the control groups showed that there was a significant difference between healthy individuals and patients in the control groups, as shown in Figure 2C(Fig. 2).

### Association between rs769446 (-427T/C) APOE gene polymorphism and risk of AD 

**Figure 3:**
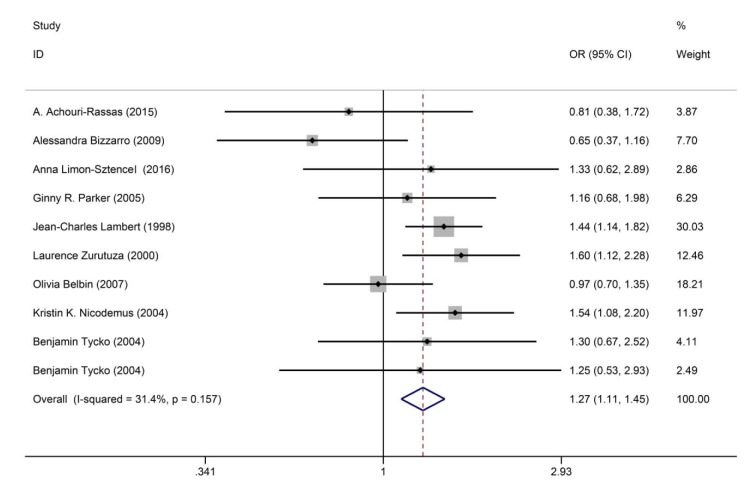
Forest plot of rs769446 polymorphism and risk for Alzheimer's disease (AD) under the allelic model

There were ten published studies that included rs769446 (-427T/C) *APOE* gene polymorphism and risk of AD. Our results found that the C allele of rs769446 variant was associated with an increase of risk of AD under the allelic model (C vs. T: OR = 1.271, 95 % CI = 1.114-1.449, *P* < 0.0001) as shown in Figure 3(Fig. 3) (References in Figure 3: Achouri-Rassas et al., 2015[1]; Bizzarro et al., 2009[10]; Limon-Sztencel, 2016[32]; Parker et al., 2005[38]; Lambert, 1998[27]; Zurutuza et al., 2000[61]; Belbin et al., 2007[8]; Nicodemus et al., 2004[37]; Tycko et al., 2004[50]). However, this variant was not associated with AD under other genetic models (CC vs. TT: OR = 0.632, 95 % CI = 0.385-1.01, *P* = 0.055; CC vs. TC: OR = 0.778, 95 % CI = 0.477-1.27, *P* = 0.316; CC+CT vs. TT: OR = 0.947, 95 % CI = 0.714-1.256, *P* = 0.708; CC vs. TT+TC: OR = 0.66, 95 % CI = 0.413-1.055, *P* = 0.083).

### Association between rs405509 (-219G/T) APOE gene polymorphism and risk of AD 

There were ten published studies that included rs405509 (-219G/T) *APOE* gene polymorphism and risk of AD. No significant association was detected between this variant and AD risk under each genetic model (T vs. G: OR = 0.947, 95 % CI = 0.738-1.216, *P* = 0.671; TT vs. GG: OR = 0.868, 95 % CI = 0.519-1.519, *P* = 0.59; TT vs. TG: OR = 0.924, 95 % CI = 0.681-1.255, *P* = 0.614; TT+TG vs. GG: OR = 0.969, 95 % CI = 0.715-1.313, *P* = 0.84; TT vs. TG+GG: OR = 0.892, 95 % CI = 0.608-1.31, *P* = 0.56).

### Sensitivity analysis and publication bias

**Figure 4:**
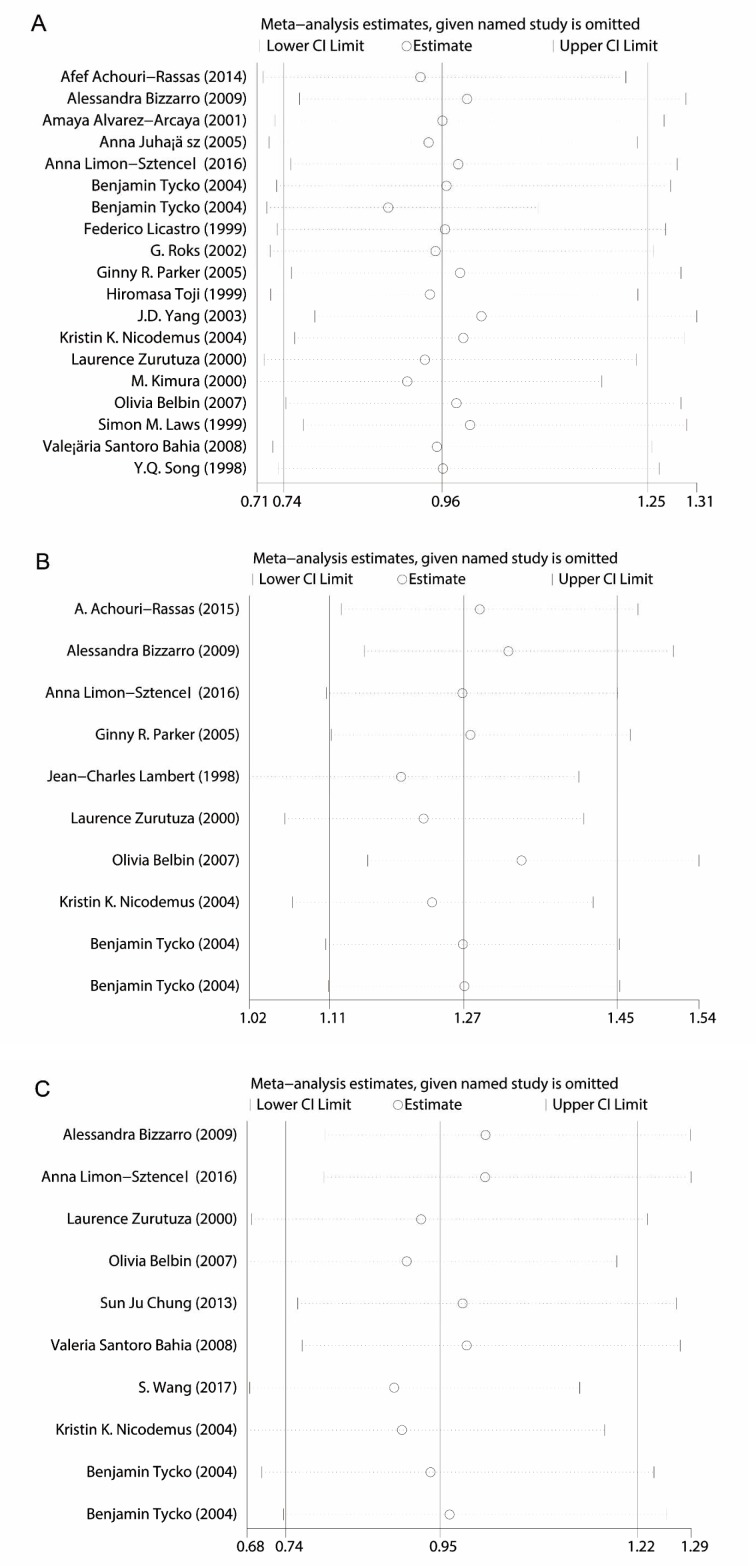
Sensitivity analysis of these three genetic polymorphisms in the promoter region of ApoE gene in AD risk (A: rs449647; B: rs769446; C: rs405509)

To observe whether the results were influenced by each of the included studies, we systematically deleted each single article at a time. The results showed that the overall results were not significantly changed, as shown in Figure 4(Fig. 4) (References in Figure 4: Achouri-Rassas et al., 2014[2]; Bizzarro et al., 2009[10]; Alvarez-Arcaya et al., 2001[3]; Juhász et al., 2005[23]; Limon-Sztencel, 2016[32]; Tycko et al., 2004[50]; Licastro et al., 1999[31]; Roks et al., 2002[42]; Parker et al., 2005[38]; Toji et al., 1999[48]; Yang et al., 2003[59]; Nicodemus et al., 2004[37]; Zurutuza et al., 2000[61]; Kimura et al., 2000[26]; Belbin et al., 2007[8]; Laws et al., 1999[30]; Bahia et al., 2008[6]; Song et al., 1998[45]; Achouri-Rassas et al., 2015[1]; Lambert et al., 1998[27]; Chung et al., 2013[13]; Wang et al., 2017[55]). 

**Figure 5:**
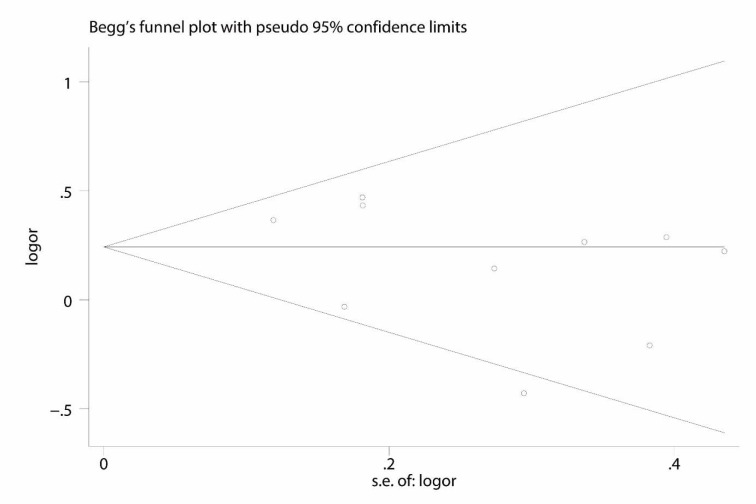
Begger's plot of rs769446 polymorphism in AD risk under the allelic model

Begg's test and Egger's test were used to assess the publication bias of the literature. Except for rs405509 variant, there was no evidence of publication bias in other genetic comparisons in our study, as shown in Figure 5(Fig. 5). These results supported that the publication bias was low in the present meta-analysis.

## Discussion

In this meta-analysis, we were able to identify 23 eligible publications on *APOE* gene polymorphisms and risk of Alzheimer's disease (AD) that satisfied the inclusion criteria of the study. The results showed that only the C allele of rs769446 polymorphism in the promoter region of *APOE* gene was associated with an increased risk of AD when compared with the T allele. 

Rs449647 and rs405509 polymorphisms of *APOE* gene were not associated with AD risk under each genetic comparison model. These results differ from the previous meta-analysis conducted by Xin et al. (2010[58]), which confirmed a significant but modest association between *APOE* promoter -491A/T and -219T/G polymorphisms and AD susceptibility.

AD is a complex disease resulting from the interaction between genetic and environmental factors. A major function of ApoE is to mediate the binding of lipoproteins or lipid complexes in the plasma or interstitial fluids to specific cell-surface receptors (Huang and Mahley, 2014[22]). Reduced levels of plasma *APOE* have been shown to be significantly correlated with reduced hippocampal size, which may reflect a component of the neuropathology of AD in elderly individuals (Teng et al., 2015[47]). 

*APOE* gene isoforms can differentially influence total serum cholesterol levels that support lipid transport and injury repair in the brain, and its genotypes are considered to be genetic risk factors for AD susceptibility. Published data supports that *APOE* ɛ4 leads to synaptic deficits and impairment in long-term potentiation, memory, and cognition (Kim et al., 2014[25]). The *APOE* ɛ4 genotype has been shown to be the strongest single genetic factor associated with cerebrospinal fluid *APOE* protein levels, and *APOE* protein levels in cerebrospinal fluid may be a useful phenotypic biomarker for AD risk (Cruchaga et al., 2012[15]). Also, serum ApoE was shown to be associated with long-term risk of AD in the general population, independent of *APOE* genotype (Wolters et al., 2016[57]). *APOE* gene expression level is a risk factor for AD irrespective of *APOE* ε4 allele status. 

Variations in levels of *APOE* have been tied to the risk and progression of AD. A previous meta-analysis has shown that *APOE* locus reached genome-wide significance in AD risk (P < 5×10^−8^) (Lambert et al., 2013[29]). The *APOE* promoter genetic variants may influence the gene transcription level, thus involving in the progression of AD. 

The -491AA polymorphism in the *APOE* gene was shown to be associated with increased plasma ApoE levels in patients with AD (Laws et al., 1999[30]). Artiga et al. (1998[5]) have shown that polymorphisms at sites -491 and -219 of the *APOE* promoter produced variations in the transcriptional activity of this gene, most likely through differential binding of nuclear proteins. Maloney et al. (2010[34]) found that the -219 and -491 polymorphic variations were significantly associated with the incidence of AD and -491 AA was significantly associated with increased risk even when stratified for the *APOE *ε4 allele. Wang et al. (2000[54]) showed that the -491 AA genotype appeared to be an independent genetic risk factor for AD. Nicodemus et al. (2004[37]) demonstrated that the -219 T allele (*P* = 0.009) was associated with increased risk of AD in age-stratified analysis in patients with AD at age of onset of 80 years or more and age-matched controls. Lambert et al. (2004[28]) found that -219 G/T promoter polymorphism influenced binding to the estrogen receptor and altered transcriptional activity in response to estrogen, possibly being involved in increased risk for AD in women bearing an ε4 allele. Bizzarro et al. (2009[10]) have confirmed the role of the -491 AA genotype as a risk factor for AD in Italy and suggested that promoter genotypes and *APOE* haplotypes might have a complex function in AD-associated genetic risk factors.

Other genetic variants may also be associated with increased risk of AD. The presence of an *APOE* ε4 allele has been shown to have a more deleterious effect on younger patients with AD when compared with older patients with AD on cognition and brain structure both in cross-sectional and longitudinal studies (Chang et al., 2014[12]). *APOE *gene status and family history of dementia and AD have been shown to be significantly associated with amyloid load; the *APOE* genotype appears to be an important driver of amyloid levels (Vemuri et al., 2013[51]). 

Some familiar generic drugs (such as gene-based, alternate approach) have known safety profiles that can deter unexpected risks. Researchers have suggested that *APOE* genotype status can be safely and effectively disclosed to individuals at high risk of AD (Scott Roberts and Green, 2015[44]). In mouse models, *APOE*-directed treatments were shown to rapidly clear β-amyloid and reverse neurological deficits in AD (Cramer et al., 2012[14]). In patients with AD, the *APOE* genotype may modify therapeutic responses (Hanson et al., 2015[19]). Studies have also identified that potential *APOE* inducer agents could be used *in vivo* for the treatment, and possibly the prevention of sporadic AD (Poirier et al., 2014[40]). 

There were several limitations to this study. First, there was high between-study heterogeneity observed, which could have affected the accuracy of the results. Second, the diagnostic criteria for AD in the patients and the condition of the controls in the retrieved publications were not consistent. Third, other factors such as environmental risk factors, gender, sex, and age, could not be considered due to lack of details in the publications. Understanding these risk factors and protective factors of AD is important for developing individualized interventions for the prevention and treatment of AD.

In conclusions, the findings of this meta-analysis showed that rs769446 polymorphism in the *APOE* gene promoter region could be a possible risk factor for AD. Future large, well-designed, multicenter, controlled clinical studies are still needed to explore further the relationship between variants of *APOE* gene promoter region and risk of developing AD.

## Conflict of interest

The authors declare that they have no conflict of interest.

## Figures and Tables

**Table 1:**
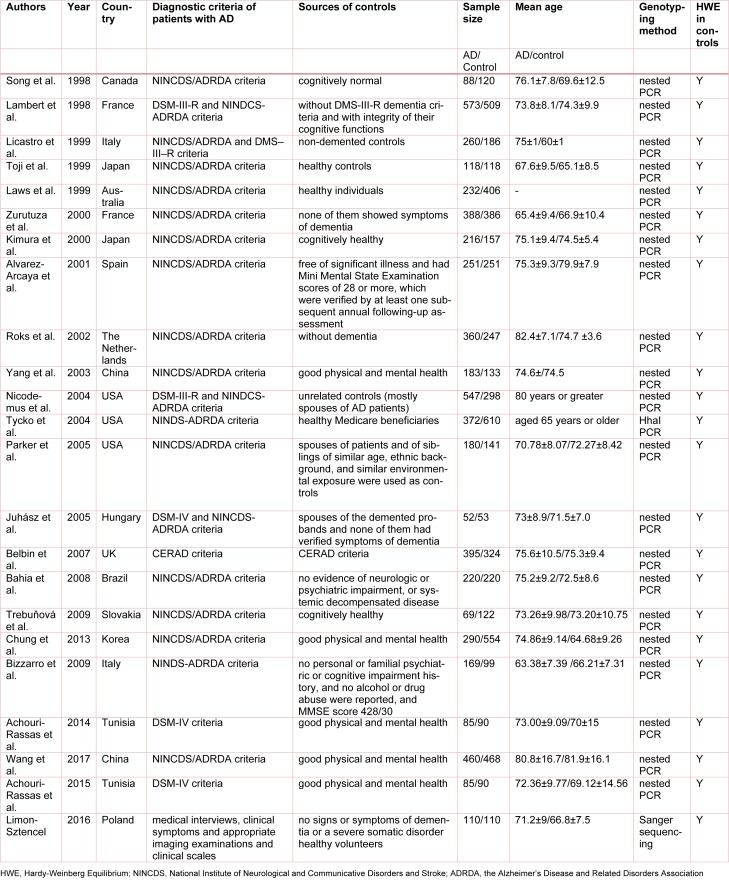
Characteristics of the included studies and patient baseline demographics

**Table 2:**
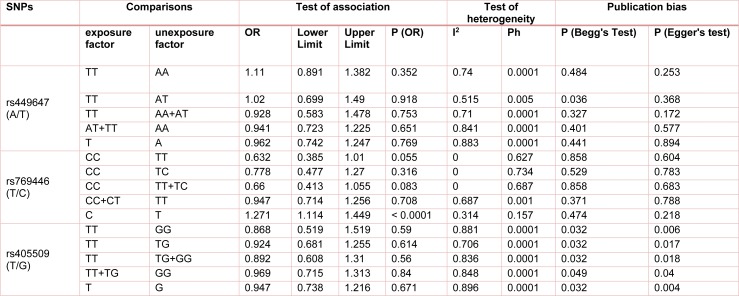
The pooled analysis between polymorphisms in the promoter region of *APOE* gene and risk of Alzheimer's disease (AD)
